# Linking Older Adults’ Daily Activities With Well-Being and Cognition: Examining Moderators and Mediators

**DOI:** 10.1093/geroni/igab046.2220

**Published:** 2021-12-17

**Authors:** Christina Roecke, Minxia Luo, Thomas M Hess

## Abstract

Increasingly more studies are showing that daily activities can be beneficial to wellbeing and cognitive abilities of older adults, but discussions about through which psychological mechanisms daily activities are associated with wellbeing and cognitive health have been scarce. This symposium, including three ambulatory assessment studies and one cross-sectional study, presents emerging theoretical hypotheses and recent empirical findings on this matter. Specifically, with 5-6 days of observations from 313 older adults, Brown and colleagues show that greater daily activity diversity is related to older adults’ higher overall cognitive functioning (executive functioning, memory, and crystallized intelligence). Hueluer and colleagues examine the moderating role of interaction modality on the relation of daily social interactions with wellbeing using data from 116 older adults over 21 days. Their results show that more face-to-face interactions — but not telephone or digital interactions — are associated with higher positive affect and lower loneliness. With data from 153 older adults over 15 days, Luo and colleagues show the mediating effect of positive affect in the association between momentary working memory performance and subsequent social activity engagement. Sharifian and colleagues show the mediating effects of solitary-cognitive activities in the association between depressive symptoms and global cognition, using cross-sectional data from 453 older adults, and also examine the moderating role of race. Finally, Tom Hess will serve as a discussant and provide an integrative discussion of the papers, informed by his extensive work on daily activities, motivation, and aging.

